# *Epitomaptasimentalae* sp. n., a new species of apodous sea cucumber from the Central Eastern Pacific coast of Mexico (Echinodermata, Holothuroidea, Apodida)

**DOI:** 10.3897/zookeys.817.29406

**Published:** 2019-01-15

**Authors:** Francisco Alonso Solís-Marín, Carlos Andrés onejeros-Vargas, Andrea Alejandra Caballero-Ochoa, Julio Adrian Arriaga-Ochoa

**Affiliations:** 1 Colección Nacional de Equinodermos “Dra. Ma. Elena Caso Muñoz”, Laboratorio de Sistemática y Ecología de Equinodermos, Instituto de Ciencias del Mar y Limnología (ICML), Universidad Nacional Autónoma de México (UNAM), Ciudad de México, C.P. 04510, México Universidad Nacional Autónoma de México México Mexico; 2 Posgrado en Ciencias del Mar y Limnología, UNAM; Av. Ciudad Universitaria 3000, C.P. 04510, Coyoacán, Ciudad de México, México Universidad Nacional Autónoma de México México Mexico; 3 Facultad de Ciencias, UNAM. Circuito exterior s/n, Ciudad de México, C. P. 04510, México Universidad Nacional Autónoma de México México Mexico

**Keywords:** Leptosynaptinae, Synaptidae, Taxonomy, Leptosynaptinae, Synaptidae, Taxonomía

## Abstract

*Epitomaptasimentalae***sp. n.** occurs in depths of 4–10 m off the Mexican Central Pacific coast. It is distinctive in having twelve tentacles, each tentacle with two or three pairs of digits and four to six sensory cups, lacking papillae or oval bumps and in reaching a maximum length of 50 mm in life.

## Abstract (es)

*Epitomaptasimentalae***sp. n.** fue recolectada a profunidades de entre 4–10 m en el Pacífico Central Tropical. Se distingue de sus congéneres por poseer doce tentáculos, cada tentáculo tiene dos o tres pares de dígitos, los cuales a su vez cuentan con cuatro a seis copas sensoriales, no posee papilas o protuberancias ovaladas sobre la superficie del cuerpo y por tener tallas desde los 4 hasta los 50 mm de largo total en ejemplares vivos.

## Introduction

Sea cucumbers of the family Synaptidae (order Apodida), while resolved as a non-monophyletic group in recent molecular analyses (Miller et al. 2017), includes the genus *Epitomapta*, a shallow water transisthmian taxonomic group of burrowing apodous sea cucumbers. The genus was created by Heding (1928) to include the previously described *Epitomaptaroseola* (Verrill, 1873) and his new species *E.tabogae* Heding, 1928. Heding based the new genus on the presence of notched rather than perforated radial pieces of the calcareous ring.

The genus is represented by three nominal species, including the new one described here. In 1952 Cherbonnier described *Epitomaptaknysnaensis* from the South African coasts but in 1989 Thandar and Rowe transferred the species into the genus *Leptosynapta* on the basis of new collections from the type locality and on the rexamination of the type material.

## Materials and methods

Specimens were collected by SCUBA diving (4–10 m depth). They were relaxed in a solution of 4% magnesium chloride and seawater. Fixation was made using 70% ethanol. Ossicles were extracted from the body wall (anterior, medium and posterior region) and tentacles. The tissue was dissolved in fresh household bleach [5–6.5%] in centrifuge tubes. After centrifugation at 1000 rpm for 10 minutes, bleach was pipetted off and the ossicles were rinsed and centrifuged with distilled water that was pipetted off afterwards. The same process was done with 70, 80, and 95% ethanol. Absolute ethanol was added to the ossicles, and finally a small aliquot was taken and placed to dry on a cylindrical double-coated conductive carbon tape stub. Then it was sputter coated with gold 2.5 kV in the ionizer Polaron E3000 for 3 minutes and photographed using a Hitachi S-2460N scanning electron microscope (SEM). Ciliated funnels were detached from the internal body wall using tweezers and dehydrated by critical point drying and placed on a carbon tape stub. Specimens were deposited at the following scientific collections: Colección Nacional de Equinodermos “Dra. Ma. Elena Caso Munoz”, Instituto de Ciencias del Mar y Limnología, Universidad Nacional Autónoma de México, Ciudad de México and Smithsonian Institution, Natural History Museum, Washington, D.C., United States.

Abbreviations used in the text:

**ICML-UNAM** Colección Nacional de Equinodermos, Instituto de Ciencias del Mar y Limnología, Universidad Nacional Autónoma de México;

**USNM** Smithsonian Institution, Washington, D.C., United States Natural History Museum.

## Taxonomy

### Order Apodida Brandt, 1835

#### Family Synaptidae Burmeister, 1837

##### Subfamily Leptosynaptinae Smirnov, 1989

**Diagnosis.**Synaptidae with 10, 11 or 12 pinnate tentacles, with one to nine digits on each side. Digits increase in size from base to tip of tentacle. Anchor plate develops from a rod which lies at a right angle to stock of developing anchor. Anchor plates with small number of holes, usually seven (6+1) in main part of the plate: six holes form a circle around a central hole. Articular end of plate usually has a “ledge” for contact with anchor keel. Anchor arms serrated, rarely naked, and without minute knobs on vertex (Smirnov 1989).

###### Genus Epitomapta

Taxon classificationAnimaliaApodidaSynaptidae

Heding, 1928

####### Emended diagnosis.

Tentacles pinnate, usually 12. Digits from two to five pairs on each side (rarely two or none). Sense organs never in the form of pigment-eyes, but occur as minute cups on inner face of stalk of tentacles. Calcareous ring well developed. The radial pieces are not perforated for the passage of nerves, but with a notch in the anterior margin. Cartilaginous ring absent. Polian vesicle usually single. Stone canal single, unbranched. Ciliated funnels are of different shapes and are attached to the body wall, not to mesenteries. The calcareous deposits in the body wall are anchors, anchor plates and miliary granules; in the tentacles large rods. Stock of anchors finely toothed, but not branched; arms usually with teeth on the outer edge; vertex smooth. Anchor plates oval or somewhat elongated, with large central hole, surrounded by six large holes, usually more or less dentate, and two large and several small smooth holes at the narrow posterior end, but without an arched bow crossing the outer surface; at the broad end there are often additional dentate holes (modified from Heding 1928).

####### Type species.

*Epitomaptatabogae* Heding, 1928 (original designation).

###### Epitomapta simentalae sp. n.

Taxon classificationAnimaliaApodidaSynaptidae

http://zoobank.org/7C6055AD-EC7E-4C8E-B702-7F31E264729D

Figs 1 , 2 , 3 , 4

####### Type material.

Holotype ICML-UNAM 5.169.0, 19 mm total length (TL), Caleta, Acapulco Bay, Guerrero, Mexico, Pacific Ocean 16°49.812'N, 99°59.062'W, 10 m depth, 8 May 2008, coll. F. A. Solís-Marín.

Paratypes: USNM 1114315, 10 specimens, same data as the holotype; ICML-UNAM 5.169.1, 11 specimens, same data as the holotype; ICML-UNAM 5.169.2, 63 specimens, Caleta, Acapulco Bay, Guerrero, Mexico, Pacific Ocean 16°49.812'N, 99°59.062'W, 10 m depth, 28 October 2006, coll. F. A. Solís-Marín, Y. Yerye, Honey-Escandón, M., A. Martínez Melo; ICML-UNAM 5.169.3, 20 specimens, Caleta, Acapulco Bay, Guerrero, Mexico, Pacific Ocean 16°49'N, 99°59'W, 4 m depth, 2 March 2006, coll. F. A. Solís-Marín, C. S. Frontana Uribe; ICML-UNAM 5.169.4, 27 specimens, Caleta, Acapulco Bay, Guerrero, Mexico, Pacific Ocean 16°49'N, 99°59'W, 9 m depth, 27 September 2006, coll. F. A. Solís-Marín, B. Urbano, M. A. Torres; ICML-UNAM 5.169.5, 5 specimens, Caleta, Acapulco Bay, Guerrero, Mexico, Pacific Ocean 16°49'N, 99°59'W, 8 m depth, 21 March 2009, coll. F. A. Solís-Marín and J.A. Díaz-Jáuregui.

####### Type locality.

Caleta, Acapulco Bay, Guerrero, Mexico, Pacific Ocean 16°49.812'N, 99°59.062'W.

####### Diagnosis.

Body wall smooth, lacking papillae or oval bumps. Tentacles 12, each with two or three pairs of digits and a terminal digit; up to six sensory cups on each tentacle. One Polian vesicle. Stone canal single, unbranched. Anchor and anchor plates of one kind, large, anchors usually exceeding 120 µm in length, plates exceeding 100 µm in length. Miliary granules numerous, in form of C-shaped rods with enlarged ends and O-shaped ossicles present only in the longitudinal muscles. Tentacle ossicles curved spiny rods with perforated ends.

####### Holotype description.

**Figure 1.**
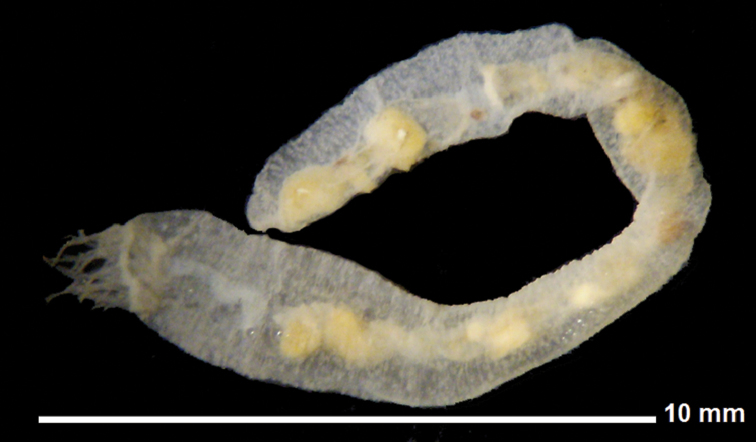
*Epitomaptasimentalae* sp. n. Holotype ICML-UNAM 5.169.0. Lateral view from preserved specimen.

**Figure 2.**
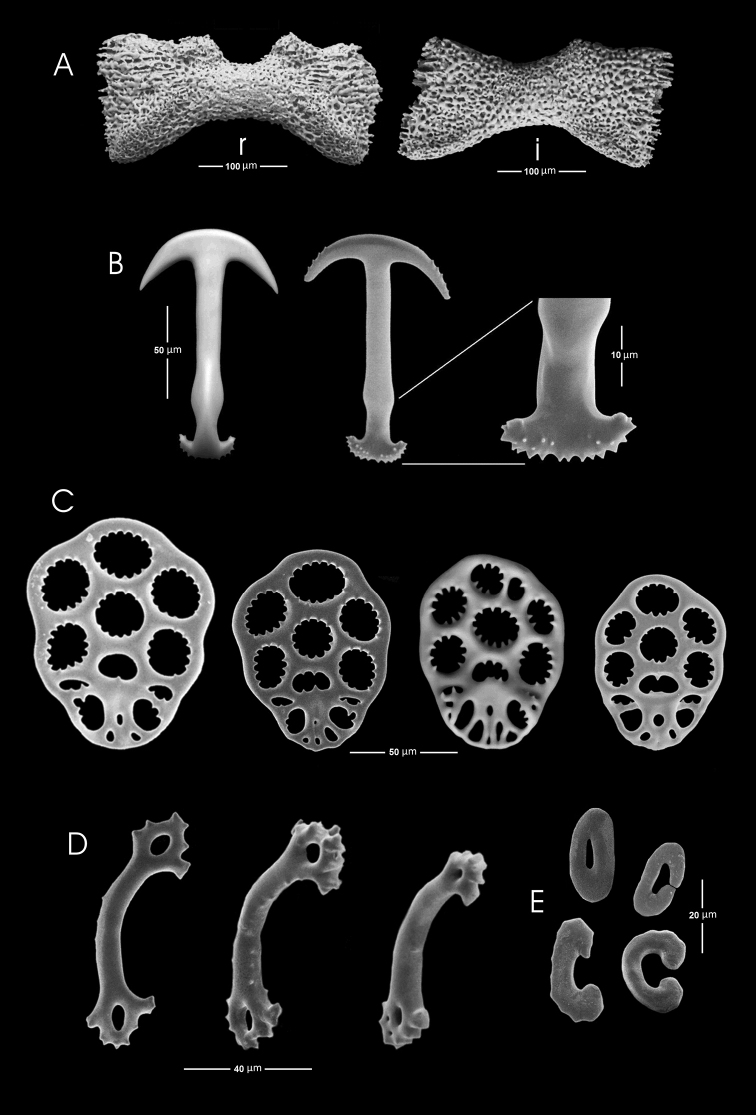
*Epitomaptasimentalae* sp. n. Holotype ICML-UNAM 5.169.0, **A** Calcareous ring, r= radial piece, i= inter-radial piece **B** Anchors from mid-body, showing the detail of the posterior part **C** Anchor plates from mid-body **D** Rods from tentacles **E** Miliary granules from the body wall.

**Figure 3.**
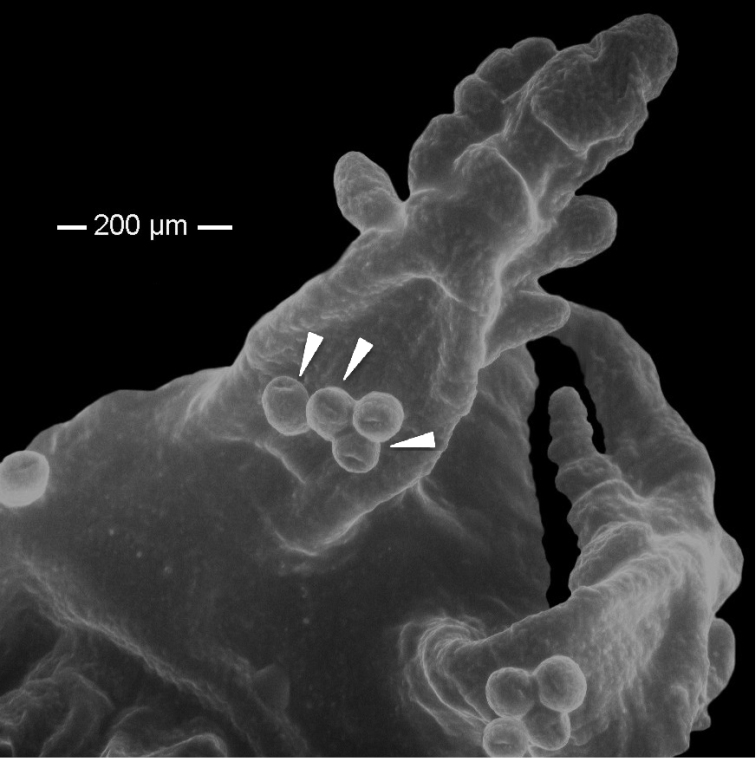
*Epitomaptasimentalae* sp. n. Holotype ICML-UNAM 5.169.0. Detail of the tentacular crown showing the sensory cups.

**Figure 4.**
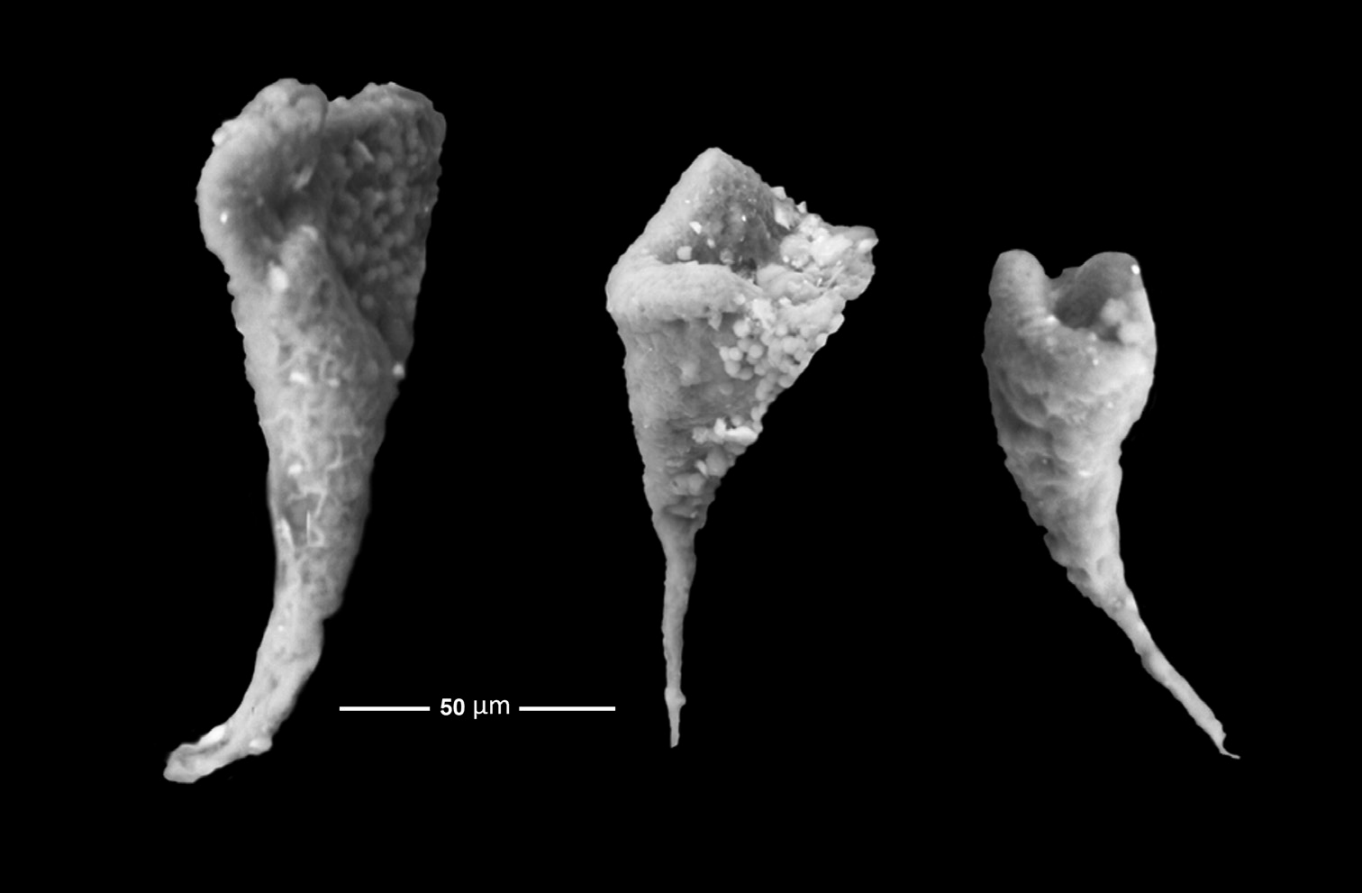
*Epitomaptasimentalae* sp. n. Paratype ICML-UNAM 5.169.3. Ciliated funnels showing their different sizes and shapes.

19 mm total length (TL).When preserved is uniformly whitish, body wall translucent when expanded (Fig. 1); color in life pink to light purple. Gonads well developed, yellowish in preserved specimens (Fig. 1). Anchors (Fig. 2B) project through body wall. Tentacles 12, each with two to three pairs of digits and a terminal digit; digits increase in length distally, and terminal digit is longest. Inner (oral) surfaces of tentacles with double row of well-developed sensory cups; up to six sensory cups on each tentacle (Fig. 3). Ciliated funnels of various shapes (Fig. 4) occur on the body wall, not on the mesenteries. There are two longitudinal rows of ciliated funnels, each row attached to one side of one longitudinal muscle. Polian vesicle single. Stone canal single, unbranched. Calcareous ring simple, well developed (Fig. 2A); the radial pieces (Fig. 2Ar) have a cavity in the central region more conspicuous than that in inter-radial pieces (Fig. 2Ai).

*Ossicles.* Body wall deposits, anchors, and anchor plates of one kind (Fig. 2B–C). Anchors and plates at anterior, middle and posterior body wall essentially similar, although developmental stages of these ossicles more numerous posteriorly; anchors of this region (in a ventral view) have the right arm slightly more elongated than the left. Anchors average 120 µm in length. Arms carry up to six conspicuous teeth. Stock unbranched, but equipped with numerous small sharp projections (Fig. 2B). Anchor plates elongated, approximately oval, with numerous toothed perforations. Anchor plates average 100 µm in length and 90 µm in greatest width (Fig. 2C). Miliary granules numerous, present only in the epithelium covering the longitudinal muscles, highly variable in shape, but generally the miliary granules tending to be enlarged; C and O-shaped bodies are distinguishable Granules up to approximately 30 µm in length (Fig. 2E). Stems of tentacles with ossicles similar to miliary granules of longitudinal muscle epithelium but tending to be slightly smaller. In tentacle digits spiny rods of up to 90 µm length, with perforated ends (Fig. 2D).

*Paratype variations.* Specimens ranges from 4–43 mm TL. Sensory cups vary in number, fewer (2–3) in smaller specimens (4–15 mm TL).

####### Ethymology.

*Epitomaptasimentalae* sp. n. is named in honor of Dr Delia Rosalba Simental Crespo, a scientist, entrepreneur and echinoderm enthusiast, who supports research programs and marine expeditions providing passion, funding, equipment, and travel support to scientists who are involved in research and conservation efforts related to the echinoderms in the Mexican marine waters.

####### Ecology.

*Epitomaptasimentalae* sp. n. occurs at 4–10 m depth, burrowed approximately 2 cm deep in in well-aerated quartz sand.

####### Reproduction.

*Epitomaptasimentalae* sp. n. is a gonochoric species; females have lecithotrophic eggs between 140 and 150 μm in diameter; ripe gonads occupy about 80% of the celomic cavity. Neither brooding nor external sexual dimorphism was observed.

####### Geographical distribution.

Known only from Caleta, Acapulco Bay, Guerrero.

## Discussion

*Epitomaptasimentalae* sp. n. is very similar to its Caribbean congener *E.roseola*, differing in the number of pairs of digits present on the tentacles (2–4 in *E.roseola* and 2–3 in *E.simentalae* sp. n.), and in the number of sensory cups per tentacle (2–5 in *E.roseola* and 4–6 in *E.simentalae* sp. n.). In addition to the geographical distribution, *E.simentalae* sp. n. is smaller (<50 mm) than *E.roseola* (30–120 mm) (Heding 1928, Miranda et al. 2015).

*Epitomaptasimentalae* sp. n. clearly differs from *E.tabogae* and *E.roseola* in lacking papillae or oval bumps all over its body wall; the number of sensory cups per tentacle (8–14 in *E.tabogae* and 4–6 in *E.simentalae* sp. nov), and in the number of pairs of digits present on the tentacles (5–6 in *E.tabogae* and 2–3 in *E.simentalae* sp. n.). *Epitomaptatabogae* is distributed throughout the Gulf of California (Solís-Marín et al. 2009) whereas *E.simentalae* sp. n. is currently known only from the Central Eastern Pacific coast of Mexico. *Epitomaptaroseola* was previously described for the Caribbean (Bermuda) (Heding 1928), and later recorded in Connecticut, Massachusetts to Florida (USA) (Hendler et al. 1995) and recently reported for the South American coast (Brazil) (Miranda et al. 2015).

The anchors of the body wall in *E.simentalae* sp. n. are similar in shape to those of *E.roseola*, but differ in size, being approximately 90–150 μm length and 70–90 μm width in *E.simentalae* sp. n. (Fig. 2B); the anchors of the posterior region of the body wall in both species are similar and can reach up to 150 μm in length and 70 μm width; anchors from the anterior end of the body wall in *E.roseola* measure almost 120 μm in length and 70 μm in width (Heding 1928), while in *E.simentalae* sp. n. they measure from 90–150 μm length and 70 μm width. On the other hand, the anchors of the Pacific *E.tabogae* are 200 μm in length and 100 μm width in the posterior region of the body, and 170 μm length and 100 μm width in the anterior region of the body (Heding 1928); *E.tabogae* has the largest anchors in this genus (Heding 1928).

In *Epitomaptasimentalae* sp. n. the anchor plates are 100 μm in length and 90 μm in width.

*Epitomaptasimentalae* sp. n. is clearly distinguished from other species of the genus in lacking papillae or oval bumps in the body wall, a character that had been used to differentiate species of the genus by various authors (see Heding 1928 and Hendler et al. 1995).

## Key to the genus *Epitomapta*

**Table:** 

1	Papillae or oval bumps present all over the body wall	**2**
–	Papillae or oval bumps absent. With 2–3 pairs of tentacle digits, each tentacle with 4–6 sensory cups. Miliary granules “C” and “O”–shaped bodies; no papillae or oval bumps present in the body wall	***E.simentalae* sp. n.**
2	Atlantic Ocean. With 7 pairs of tentacle digits, each tentacle with 2–5 sensory cups. Anchors of body wall exceed 120 μm in length (up to 150 μm). Miliary granules in the shape of small oval rings and very few C–shaped bodies	*** E. roseola ***
–	Pacific Ocean. With 5–6 pairs of tentacle digits, each tentacle with 8–14 sensory cups. Anchors of body wall exceed 120 μm in length (up to 200 μm). Miliary granules in the shape of oval rings and very few C–shaped bodies	*** E. tabogae ***

## Supplementary Material

XML Treatment for Epitomapta

XML Treatment for Epitomapta simentalae
